# Corneal Allogenic Intrastromal Ring Segments: A Literature Review

**DOI:** 10.3390/jcm14041340

**Published:** 2025-02-18

**Authors:** Issac Levy, Ritika Mukhija, Mayank A. Nanavaty

**Affiliations:** 1 Sussex Eye Hospital, University Hospitals Sussex NHS Foundation Trust, Brighton BN2 5BF, UK; issac.levy@nhs.net (I.L.); ritika.mukhija@nhs.net (R.M.); 2 Ophthalmology Department, Rabin Medical Center, Petach Tikva 4941492, Israel; 3 Faculty of Medicine, Tel Aviv University, Tel Aviv 6997801, Israel; 4 Brighton & Sussex Medical School, University of Sussex, Falmer, Brighton BN1 9PX, UK

**Keywords:** keratoconus, ectasia, ring segments

## Abstract

**Background:** Corneal allogenic intrastromal ring segments (CAIRSs) offer a novel, biocompatible alternative to synthetic intracorneal ring segments (ICRSs). This review aims to evaluate the clinical outcomes of CAIRS. **Methods:** Inclusion criteria were studies with a minimum of 20 eyes and six months of follow up. The primary outcome measure was uncorrected distance visual acuity (UDVA). The secondary outcomes were a change in corrected distance visual acuity (CDVA), spherical equivalent (SE), mean keratometry (K-mean), maximum keratometry (K-max), K1, K2, and pachymetry. **Results:** The primary outcome UDVA improved from 0.83 ± 0.15 to 0.40 ± 0.08 logMAR (*p* = 0.01), while CDVA improved from 0.52 ± 0.22 to 0.19 ± 0.09 logMAR (*p* = 0.01). K-max decreased from 57.8 ± 1.09 D to 53.57 ± 2.66 D (*p* < 0.01), and K-mean reduced from 49.27 ± 0.28 D to 45.30 ± 1.46 D (*p* < 0.01). An average of 84.92% ± 11.4% of eyes had an improvement in UDVA. No major complications or significant visual acuity deterioration were reported. **Conclusions:** CAIRSs serve as an alternative to synthetic ICRSs and even corneal transplantation in some cases. They represent a safe, effective, and biocompatible promising advancement in corneal ectasia management to improve visual acuity and corneal topography with minimal complications.

## 1. Introduction

Corneal ectasia is a non-inflammatory disorder characterized by progressive thinning and steepening of the cornea. This results in irregular astigmatism, visual distortion, and potential vision loss. The most common conditions associated with corneal ectasia include keratoconus, pellucid marginal degeneration, and post-refractive surgery ectasia. These conditions can progress over time, making early diagnosis and treatment essential for preserving visual acuity [1,2].

Current management strategies for corneal ectasia can be divided into two arms: the first aims to stabilize the cornea and prevent further progression, and the second aims to improve the visual acuity [3]. In the early stages, conservative options include close follow-up and refractive correction with spectacles or contact lenses (soft/rigid gas-permeable (RGP)/scleral) [3]. As ectasia progresses and the cornea becomes increasingly irregular, fitting contact lenses becomes more challenging. Patients may experience intolerance to contact lenses and may need surgical interventions [3,4]. Corneal cross-linking (CXL) is a commonly used intervention, which strengthens the corneal stroma through a photochemical reaction between riboflavin (vitamin B2) drops and ultraviolet A light exposure. Studies have shown that CXL can halt disease progression and, in some cases, even improve corneal curvature, making it an effective treatment for mild to moderate ectasia [3,5,6,7].

For patients with significant corneal irregularity, intrastromal corneal ring segments (ICRSs) provide an alternative option; these rings are surgically implanted to reshape the cornea, effectively reducing astigmatism and flattening the steep areas, which improves visual acuity [3,8,9,10,11]. Synthetic ICRSs, such as polymethyl methacrylate (PMMA) segments, have shown efficacy in reshaping the cornea, although complications like extrusion and infection can occur. The main reasons for ICRS use are functional failure, refractive failure, keratoplasty surgery due to poor visual performance and anatomic failure. Spontaneous extrusion can occur (one large study on 1644 eyes reported ~2%). Other reasons are suspected infection, corneal melting and corneal perforation [12,13]. In this review, ICRSs will refer specifically to synthetic ring segments. In more advanced cases or when corneal transparency is compromised, corneal transplantation may be considered a deep anterior lamellar keratoplasty (DALK) or penetrating keratoplasty (PK) [3]. Both procedures can offer significant visual improvement but carry graft rejection, infection, and prolonged recovery time risks [14,15,16].

The use of corneal allogenic intrastromal ring segments (CAIRSs) is a surgical technique introduced as an innovative treatment for keratoconus and corneal ectasia [17]. The procedure entails implanting ring segments crafted from donor corneal stromal tissue into stromal channels within the patient’s cornea. CAIRSs were developed as a natural alternative to synthetic intrastromal ring segments to improve corneal stability and decrease astigmatism while reducing the risks associated with synthetic implants. They provide a less invasive option for patients with advanced keratoconus over corneal transplant, especially when other treatments, such as collagen crosslinking or contact lenses, are either inadequate or unsuitable. Unlike synthetic ICRSs, which may carry risks of extrusion, inflammation, or infection, CAIRSs, being allogenic, offer better biocompatibility and integration within the corneal stroma, potentially leading to more stable long-term outcomes. Additionally, CAIRSs can be customized in terms of size and shape to better fit individual patient anatomy and disease progression, allowing for a more tailored approach to treatment. However, there are limited clinical data because this is a relatively recent advancement in managing keratoconus and corneal ectasia. This review aims to consolidate the knowledge regarding CAIRSs and evaluate their clinical outcomes. By synthesizing the available literature, we seek to provide a clearer understanding of its potential benefits, limitations, and future directions in the field of corneal ectasia management.

## 2. Methods

**Figure 1:**
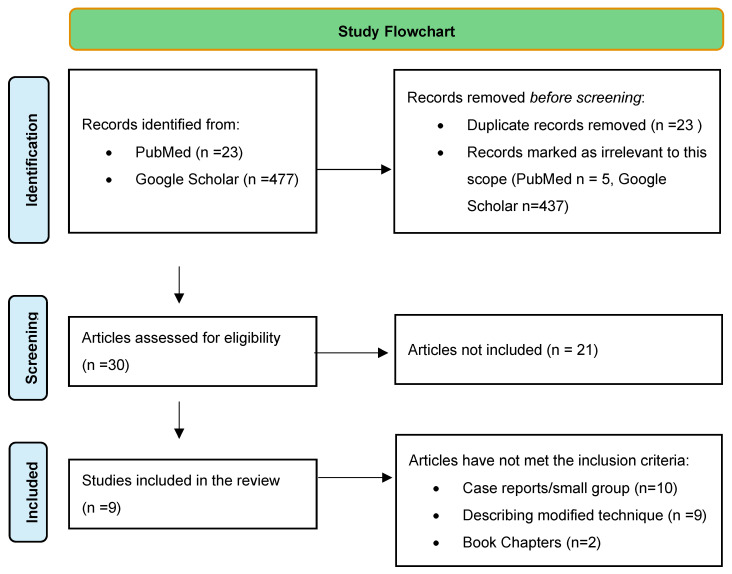
Study Flowchart.

A comprehensive literature search was conducted in PubMed/Medline and Google Scholar to identify studies regarding CAIRSs in English. We used the term “corneal allogenic intrastromal ring segments”. We did not find any articles published in languages other than English. The titles and abstracts resulting from the searches were reviewed. A full-text copy of all potentially relevant studies was reviewed for eligibility, and only those assessing corneal allogenic intrastromal ring segments were included in the study. Inclusion criteria were studies reporting clinical outcomes and/or complications after a CAIRSs procedure with or without corneal collagen crosslinking (CXL) with at least 20 eyes and a minimum follow-up of 6 months. Twenty-one articles were excluded from the study; the flowchart describes the process (Figure 1) (Supplementary File S1). Data from the final follow-up was used for analysis. The primary outcome measure was uncorrected distance visual acuity (UDVA). The secondary outcomes were a change in corrected distance visual acuity (CDVA), spherical equivalent (SE), mean keratometry (K-mean), maximum keratometry (K-max), K1, K2, and pachymetry. Data were inputted on a spreadsheet by one reviewer and re-checked and validated by a second reviewer. Statistical analysis was performed with a dependent samples T-test, and the level of statistical significance was set at *p* < 0.05. All visual acuity data were standardized by converting them into logMAR format when originally presented in Snellen or decimal formats. This conversion allowed for uniformity in the measurement scale, enabling more precise statistical interpretation and comparison across datasets.

## 3. Results

Nine studies were found eligible and were evaluated in this review; these were published between 2018 and 2024. A total of 303 patients and 389 eyes, of which 102 eyes had CXL, were included [17,18,19,20,21,22,23,24,25]. The mean follow-up period was between 6 and 23 months. Table 1 shows the demographics and results of the included studies. These included prospective [17,20,25] and retrospective [17,18,19,20,21,22,23,24] designs, focusing on keratometry, tomography, visual acuity, and corneal stability outcomes.

CAIRSs is an evolving technique, and no standardized criteria have been established yet leading to variability in surgical approaches and patient selection. CAIRSs principle is based on the Barraquer thickness law, which indicates that the outcome obtained is directly proportional to the segment’s thickness while being inversely proportional to its diameter [17]. Table 2 describes in detail each study’s inclusion and exclusion criteria and surgical technique.

The studies reviewed used femtosecond laser to create stromal tunnels for CAIRSs implantation, with inner diameters ranging from 4.0 to 6.5 mm and outer diameters from 6.8 to 8 mm at an approximately 35–50% corneal depth. All studies included patients with keratoconus in varied stages or post-laser refractive surgery ectasia, with a minimum corneal thickness of 320–400 μm. Some studies specifically included patients who had unsatisfactory visual outcomes with spectacles and were either reluctant to use or intolerant to rigid gas-permeable lenses [18,19,21]. In three studies, corneal stromal rings were prepared intraoperatively by the surgeon using the Jacob CAIRSs trephine and subsequently dehydrated for 20–45 min to facilitate implantation [17,18,20,21]. Other studies utilized pre-prepared KeraNatural^®^ (VisionGift, Boston, MA, USA) corneal stromal ring segments [19,22,23,25]. Barraquer thickness law states that corneal flattening is achieved by adding tissue to the periphery and that the outcome achieved is directly proportional to the thickness of the segment and inversely proportional to its diameter [26]. No standardized nomogram was universally applied; most studies customized implantation based on individual topographic features. Bteich et al. [18,24] employed a nomogram modified from their previous work on PMMA ICRSs, whereas Yucekul et al. [23], Haciagaoglu et al. [19] and Keskin Perk et al. [25] applied the Istanbul nomogram [19].

All the reviewed studies reported statistically significant improvements in key clinical parameters, including keratometry values and visual acuity following CAIRSs implantation.

### 3.1. Primary Outcome

The mean pre-operative uncorrected distance visual acuity (UCDVA) was 0.83 ± 0.15 logMAR; this improved to 0.40 ± 0.08 logMAR (*p* = 0.01). Four studies reported the percentage of patients experiencing an increase in UDVA, with a mean improvement rate of 84.9% ± 11.4% [17,18,21,22,25].

### 3.2. Secondary Outcomes

CDVA showed notable enhancement, with mean values improving from 0.52 ± 0.22 to 0.19 ± 0.09 logMAR (*p* = 0.01). SE improved significantly from the mean value of −7.09 D to −2.34 D (*p* < 0.01). Keratometry findings indicated a reduction in corneal curvature, with the mean K-max decreasing from 57.8 ± 1.09 to 53.57 ± 2.66 (*p* < 0.01) and the mean K-mean decreasing from 49.27 ± 0.28 to 45.30 ± 1.46 (*p* < 0.01). The mean K1 value decreased from 47.30 ± 0.51 D pre-operatively to 43.31 ± 0.90 D postoperatively, reflecting an average reduction of 3.99 D (*p* < 0.01). Similarly, the mean K2 value decreased from 51.49 ± 0.24 D pre-operatively to 47.05 ± 1.48 D postoperatively, corresponding to an average reduction of 4.44 D (*p* < 0.01).

Corneal pachymetry values remained stable, with the mean pre-operative thickness of 443 μm showing no significant change postoperatively (442 μm, *p* = 0.93). No major complications were reported across all studies. One study reported one case of graft dislocation towards the implantation site and dehiscence at the tunnel entrance, which was observed in the first postoperative week, and this patient’s graft was repositioned successfully [22]. Kirgiz et al. [21] reported that one patient complained of halo and glare at night postoperatively. Lastly, two studies reported one patient who lost one line of CDVA three months postoperatively [18,24].

Across studies, the improvement in CDVA ranged from no improvement in 0% to 37.5% (mean of 10.6% ±12.7) of the patients to the majority of patients, to improvement in 62.5–100% (mean of 88.5% ±12) of patients, with improvements from one to seven lines on a visual acuity chart.

## 4. Discussion

This narrative review evaluated the outcomes of CAIRSs in the management of keratoconus. The findings demonstrate that using CAIRSs significantly improves corneal topographic parameters and, hence, visual acuity, both uncorrected and corrected. These results are consistent with prior reports of the efficacy of natural corneal implants in reshaping and stabilizing the cornea. UDVA and CDVA improved in mild and severe keratoconus cases, reflecting enhanced corneal regularity and optical quality. Cases with decentered cones or prior CXL also showed favorable outcomes, suggesting the effectiveness of CAIRSs across varying keratoconus presentations. Topographic parameters, such as keratometry and corneal symmetry, showed statistically significant improvements across all studies. The average reduction in steep keratometry (Kmax) ranged from 2 D to near 11 D, reflecting corneal flattening and stabilization.

In cases where progression is anticipated, CAIRSs can be combined with CXL [17,18,19,20,21,22]. The procedure may be performed as an independent treatment in older patients who require only the regularization of corneal topography. CXL strengthens the biomechanical integrity of the cornea by inducing collagen cross-links, halting the progression of the disease. At the same time, CAIRSs addresses the corneal shape by reducing irregularity and improving visual acuity. The timing of corneal cross-linking (CXL) varied, with some studies including patients who had previously undergone CXL [21,22], others performing it immediately after CAIRSs implantation [17,20], deferring it for 6 months [19], or those not performing CXL at all [18]. Interestingly, Yucekul et al. [23] demonstrated that eyes without CXL achieved a statistically significant gain in CDVA compared to those with CXL (three lines vs. two lines). Non-CXL eyes also exhibited a greater reduction in K1 and K-mean. This phenomenon can be attributed to the stiffening effect produced by the CXL procedure, which suggests that the CXL process of stiffening the corneal stroma may limit the flexibility and the flattening effect of the stromal rings. However, there were no statistically significant differences between the groups in uncorrected distance visual acuity (UDVA), spherical equivalent (SE), K2, Kmax, or pachymetry (*p* > 0.05) [23]. On the other hand, Kirgiz et al. [21] reported that when comparing patients with and without previous CXL, there was no statistically significant difference between the two groups in terms of pre-and postoperative UCVA, CDVA, K-mean, and SE (*p* > 0.05). 

The innovation of CAIRSs was driven by the will to find another therapeutic module for corneal ectasia and the limitations of synthetic intrastromal ring segments [17]. While effective in reducing corneal curvature, these synthetic implants carry risks such as extrusion, infection, and intolerance due to their synthetic nature [13]. By contrast, CAIRSs utilizes biocompatible donor corneal tissue, which reduces the risk of rejection and integrates better with the host tissue. Hence, the risk for implant extrusion is postulated to be lower than that of synthetic alternatives (up to 30%). In our review, there was no case of stromal ring extrusion. The overall safety profile of CAIRSs was favorable, with most patients achieving stable and improved outcomes without significant long-term complications. There are two major causes of ICRSs extrusion: superficial implantation of a segment and a segment placed too close to the incision [12]. As a general rule, the thickness of the implanted synthetic ICRSs should not be more than 50% of the corneal thickness in the ring track. Moreover, the incision depth should preferentially be set at 80% of the corneal thickness. Another advantage of CAIRSs is that it can be implanted in thinner corneas and at shallower depths due to reduced risk for melt or necrosis and better biointegration [27,28]. CAIRSs can also be used to exchange synthetic ICRSs in case of extrusion or intrusion [29,30].

Although the studies in this review employed femtosecond lasers to create tunnels for implantation, it is important to highlight that the CAIRSs technique can also be performed manually through manual channel creation and segment insertion. One study reported successful outcomes in all 26 eyes treated, with no significant complications observed during the short follow-up period. This manual approach provides a viable option in settings where femtosecond technology is unavailable or unaffordable [31].

In five out of nine studies, two nomograms were used for CAIRSs; these nomograms are intended to guide surgeons in selecting the appropriate ring segment shape, size and thickness, and positioning based on individual patient parameters such as keratometry and corneal thickness. Both nomograms showed comparable non-superior results with all other studies. Studies using the Istanbul nomogram [19,23,25] have shown mean improvements of 0.43 ± 0.09 logMAR (*p* = 0.48) and 0.35 ± 0.10 logMAR (*p* = 0.38) in UDVA and CDVA, respectively. Bteich et al. [18,24], who used a nomogram modified from their previous work on PMMA ICRSs, showed a mean improvement of 0.39 ±0.02 logMAR (*p* = 0.06) and 0.38 ± 0.05 logMAR (*p* = 0.22) in UDVA and CDVA.

The keratometry values were also comparable, with no one approach being better than the others. This emphasizes that CAIRS should be tailored and probably have a learning curve for each surgeon based on factors such as CAIRSs size, shape, tunnel depth, and length. Another new approach, customizing CAIRSs, may offer advantages for treating keratoconus, particularly in cases with decentered or asymmetric cones. CAIRSs allow for the creation of segments tailored to each patient’s cornea’s topographic and refractive characteristics. Each segment can have different arc lengths and thicknesses along the segment, leading to better corneal regularization and, thus, visual outcomes. The concept is to add more tissue volume to areas of the meridian that require greater flattening while providing less volume where less flattening is necessary [20,32]. Soosan et al. were the first to show a significant difference in the flattening obtained at the different zones as planned before surgery; this was accomplished using a specialized, dome-shaped customizer template instrument (Jacob CAIRSs customiserTM, Epsilon Instruments, Austin, TX, USA) designed to enable precise customization. This convex titanium disk features circular optical zones ranging from 3 to 12 mm, as well as radial clock hours and the 12 principal meridians marked upon it. The CAIRSs is aligned with the intended optical zone of the CAIRSs customizer according to the plan established for the eye [17]. Still, there was no significant difference between the height and width of the segments measured by Anterior segment OCT. However, customized CAIRSs can be more time-consuming, necessitate extra tools, and might require a femtosecond laser to be even more precise in this segment [33,34].

Synthetic ICRSs have shown significant efficacy in improving visual acuity and reducing corneal curvature in keratoconus and post-laser refractive surgery ectasia. Various studies reported significant improvements in UDVA and CDVA, along with reductions in keratometric values [35]. ICRSs, although effective, has more risks of complication, as we discussed in the introduction, and can have only limited combinations of shapes and sizes, resulting in having to limit the choice to the best possible one, while CAIRSs offers infinite possibilities to customize, allowing true personalization to each patient. Our review of 389 eyes from studies between 2018 and 2024 found clinical improvement in visual acuity and keratometry comparable to those under ICRSs. Additionally, a recent study comparing CAIRSs and synthetic ICRSs using propensity score matching found both methods resulted in significant improvements in CDVA, refractive astigmatism, and keratometry values [27]. However, CAIRSs demonstrated a higher percentage of eyes gaining two or more Snellen lines of CDVA (60% vs. 31.58%, *p* = 0.04) [27].

Immunologically, CAIRSs is thought to carry low rejection risks due to the immune-privileged status of the cornea and the absence of vascularization in the implantation area. However, long-term immunological safety data are still sparse and warrant further investigation. A potential risk of stromal rejection is associated with this procedure. Still, understandably, the risk is lower than that of DALK, which has a low risk on its own due to the reduced amount of stromal transfer, absence of epithelial or endothelial transfer, early repopulation of tissue by keratocytes on all sides, and implantation only in the mid-peripheral zone with complete sparing of the pupil axis. Using processed tissue allows for further deantigenization [36,37].

The limitations of this review are primarily characterized by the restricted availability of data and their heterogeneity, a common challenge associated with emerging surgical methods. Another significant issue is the lack of standardization and consistency in procedures. Variations in tissue preparation, thickness, and preservation methods can result in inconsistent outcomes across various centers. There is a lack of long-term follow-up, as we have in the case of synthetic ICRSs. While the short-to-medium-term results are promising, there are limited long-term data regarding the durability of outcomes, potential complications, and tissue integration. Larger studies are needed for more standardized and high-quality data. The evidence supporting CAIRSs is based on a few studies, often with small sample sizes and variable methodologies, making it difficult to generalize findings. To promote the broader adoption and optimization of CAIRSs, it is essential to address these limitations through standardized protocols and long-term clinical studies. This review highlights CAIRSs as a safe, effective, and biocompatible alternative to synthetic intrastromal ring segments for keratoconus management. It offers significant improvements in visual acuity and corneal topography with minimal complications.

## Figures and Tables

**Table 1:** Details of included studies.

Study	Num of Eyes	Mean Age (Years)	Follow Up (Months)	Preoperative UDVA (logMAR)	Postoperative UDVA (logMAR)	Preoperative CDVA (logMAR)	Postoperative CDVA (logMAR)	Preoperative Kmax (Diopters)	Postoperative Kmax (Diopters)	Preoperative Mean K (Diopters)	Postoperative Mean K (Diopters)
Jacob S, 2018 [17]	24	N/A	11.58 ± 3.6	0.65 ± 0.79	0.443 ± 0.65	0.22 ± 0.67	0.1 ± 0.72	55.35	52.55	49.58	48.1
ich Y, 2023 [18]	52	31.2 ± 13.6	6.9 ± 5.2	0.97 ± 0.47	0.56 ± 0.25	0.56 ± 0.19	0.22 ± 0.21	58.09	52.33	48.92	44.32
Kirgiz A, 2024 [21]	23	29.3 ± 7.2	6	1.09 ± 2	0.397 ± 1.3	0.6 ± 0.95	0.16 ± 1.04	57.65	46.96	49.86	42.4
Nacaroglu SA, 2023 [22]	65	29.5 ± 7.3	12	0.91 ± 0.5	0.36 ± 0.25	0.87 ± 0.2	0.36 ± 0.15	59.2	55.63	49.23	45.23
Yucekul B, 2024 [23]	30 With CXL	29 (median)	6	0.69 ± 0.76	0.35 ± 0.55	0.55 ± 0.69	0.26 ± 0.58	57.22	54.11	49.46	46.34
	37 without CXL	24 (median)		0.76 ± 0.79	0.3 ± 0.65	0.5 ± 0.74	0.16 ± 0.16	58.38	55.66	49.11	44.45
Haciagaoglu S, 2023 [19]	44	30.32 ± 7.43	6	0.69 ± 0.74	0.34 ± 0.58	0.53 ± 0.69	0.25 ± 0.58	57.56	55.4	49.01	45.71
Jacob S, 2023 [20]	32	N/A	12 or more	0.66 ± 0.95	0.32 ± 0.61	0.11 ± 0.72	0.05 ± 0.76	58.9	54.95	49.07	45.63
Bteich Y, 2024 [24]	20	N/A	12	0.89 ± 0.33	0.52 ± 0.16	0.57 ± 0.18	0.15 ± 0.18	57.21	52.85	49.3	45.23
Keskin Perk, 2024 [25]	49	29.04 ± 8.13	23± 13.46	0.96 ± 0.5	0.41 ± 0.34	0.72 ± 0.47	0.22 ± 0.19	58.51	55.31	49.17	45.68

UDVA = uncorrected distance visual acuity; CDVA = corrected distance visual acuity; Kmax = maximum keratometry; Mean K = mean keratometry.

**Table 2:** Inclusion and exclusion criteria of included studies and the surgical techniques.

Study	Inclusion Criteria	Exclusion Criteria	CAIRSs Size	Depth of Implantation	Corneal Tunnels Position
Jacob S, 2018 [17]	-Amsler–Krumeich stages 1–4 -Progression in keratoconus (increase in Kmax or steepest K > 0.75 D in the preceding 6 months.	-Age > 35 years -Severe allergies autoimmune and immunodeficiency syndromes -Previous viral keratitis -Corneas steeper than 68.00 D -Central or paracentral scarring -Corneal thickness < 320 μm -History of prior corneal/intraocular surgery.	-Outer diameter of 7.5 mm, an inner diameter of 6.7 mm, and a groove width of 0.40 mm	-50% depth of the minimum pachymetry in the 7 mm optical zone	-Inner diameter of 6.5 mm and outer diameter of 8 mm. -In patients with CDVA of better than 20/40, the incisions were positioned at the refractive steep axis in case of a difference of greater than 15° between the refractive and topographical astigmatic axes.
Bteich Y, 2023 [18]	-Patients with keratoconus or corneal ectasia after laser vision correction who had unsatisfactory visual results with spectacles and were reluctant to have or intolerant to rigid gas-permeable lenses.	-Corneal opacity -Previous intraocular surgeries -Viral keratitis -Autoimmune and connective tissue diseases -Follow-up period of <3 months.	-500 or a 750 μm ring of stromal tissue, which was then bisected into two equal semicircular segments. -Dehydrate for 45 min in a room with 35% to 45% humidity.	Depth ranging from 250 to 300 μm. For patients with ectasia after laser vision correction, the flap depth was determined on OCT at a diameter of 6 to 8 mm. The target tunnel depth was at least deeper than the deepest point of the flap interface plus three times the standard deviation of the laser tunnel depth (7 μm), i.e., 23, with an additional safety margin of 50 μm, ensuring a low chance of intersection with the flap.	5/6 mm inner diameter and 6.8/7.8 mm outer diameter, 900 μm tunnel width.
Kirgiz A, 2024 [21]	-Age > 18 years old -Keratoconus -Unable to achieve a satisfactory level of visual acuity with either spectacles or contact lenses. -Corneal hickness > 350 μm -contact lenses intolerance -No progression of keratoconus for at least 1 year of follow-up, and at least 1 year of follow up after corneal cross-linking (if performed -CAIRSs implantation without previous CXL was performed in patients over 35 years of age with no progression of keratoconus during at least 2 years of follow-up.	-History of prior corneal/intraocular surgery. -Corneal scarring, previous hydrops, active severe allergy or allergic conjunctivitis, breastfeeding or pregnancy. -At least 12 months after CXL.	-2 mm wide 360 degrees ring-shaped -Dehydrated for 20 min.	-Depth of 200 μm.	-Inner diameter to 4.50 mm, and the outer diameter to 7.75 mm.
Nacaroglu SA, 2023 [22]	-Age > 18 years old -Keratoconus -CDVA < 0.3 LogMAR and contact lens intolerance or who did not prefer to use contacts.	-History of a previous corneal/intraocular surgery -Post-LASIK corneal ectasia -Central or paracentral scaring -History of viral keratitis -Central corneal thickness < 400 μm	Prepared CAIRSs by KeraNatural^®^, arc length of approximately 160°	Depth of 35% of the minimum pachymetry in a 7 mm central optical zone.	Inner diameter of 4 mm and an outer diameter of 7.5 mm
Yucekul B, 2024 [23]	-Age > 20 years old -Contact lens intolerance -Corneal thickness > 400 μm at the implantation area. -Asymmetric non-central cones -Contact lens intolerance	-History of a previous corneal/intraocular surgery -Central or paracentral scaring -History of viral viral keratitis, glaucoma or any retinal disease -Autoimmune and connective tissue diseases and pregnant or lactating.	Prepared CAIRSs by KeraNatural^®^, arc length of approximately 160°	Depth of 35% of the minimum pachymetry in a 7 mm central optical zone.	Inner diameter of 4 mm and an outer diameter of 7.5 mm
Jacob S, 2023 [20]	-Amsler–Krumeich stages 1–4 with pericentral or paracentral decentered cones that showed gradation of keratometry values, with one side being steeper than the other.	-Severe allergies, autoimmune and immunodeficiency syndromes -Previous viral keratitis -Central or paracentral scarring -Corneal thickness < 320 μm -History of prior corneal/intraocular surgery.	-Outer diameter of 7.5/8.75 mm, inner diameter of 6.5/8 mm	-50% depth of the minimum pachymetry in the zone of implantation up to a maximum depth of 280 μm	-Inner diameter of 4.6 mm and tunnel width of approximately 1.5 mm.
Bteich Y, 2024 [24]	-Stable keratoconus preoperatively. -Patients with inadequate visual results with spectacles or were intolerant to rigid-gas permeable lenses.	-Corneal opacity -Previous intraocular surgeries -Viral keratitis -Autoimmune and connective tissue diseases -Follow-up period of less < 3 months.	-Outer diameter of a segment can vary up to 9 mm while the internal diameter would vary up to 8 mm for segments of 500 mm size and up to 7.5 mm for the 750 mm segments. -Dehydration	-The tunnel depth ranged from 250 to 300 mm and 700 to 900 mm in width with.	The optical zone was 6.00 mm.
Keskin Perk, 2024 [25]	-Age > 18 years old -Progressive keratoconus. -Amsler–Krumeich stages 1–3 -CDVA < 0.3 LogMAR and contact lens intolerance -Corneal thickness > 350 μm -Absence of additional ocular diseases other than keratoconus.	-Central or paracentral scaring. -History of viral keratitis -Severe dry eye -History of prior corneal/intraocular surgery -Autoimmune and connective tissue diseases and pregnant or lactating patients -Patients who had previously undergone crosslinking treatment or who had a double-ring segment implanted.	Prepared CAIRSs by KeraNatural^®^, arc length of approximately 160°	-The tunnel depth ranged from 200 to 250 μm (approximately 35–40% depth).	Inner diameter of 4 mm and an outer diameter of 7.5 mm

## Supplementary Materials

The following supporting information can be downloaded at: https://www.mdpi.com/article/10.3390/jcm14041340/s1, File S1: Excluded studies from the review.
