# Supplementary material for: Corneal Allogenic Intrastromal Ring Segments: A Literature Review
Source: J Clin Med. 2025 Feb 18;14(4):1340. doi: 10.3390/jcm14041340 (PMC11856834; doi:10.3390/jcm14041340)
Supplement: Supplementary file 1 [file jcm-14-01340-s001.zip › jcm-3473896-Excluded studies.pdf]

Supplementary file S1:
Excluded studies from the analysis:

1. Corneal Allogenic Intrastromal Ring Segments (CAIRS) Versus Synthetic Segments: A Single Segment Comparative Analysis Using Propensity Score Matching. Asfar KE, Bteich Y, Mrad AA, Assaf JF, Jacob S, Hafezi F, Awwad ST. J Refract Surg. 2024 Nov;40(11):e863-e876. doi: 10.3928/1081597X-20241002-02. Epub 2024 Nov 1. PMID: 39530978 Free article. https://pubmed.ncbi.nlm.nih.gov/39530978/

2. Management of Anterior Stromal Necrosis After Polymethylmethacrylate ICRS: Explantation Versus Exchange With Corneal Allogenic Intrastromal Ring Segments.Kozhaya K, Mehanna CJ, Jacob S, Saad A, Jabbur NS, Awwad ST. J Refract Surg. 2022 Apr;38(4):256-263. doi: 10.3928/1081597X-20220223-01. Epub 2022 Apr 1. PMID: 35412922 https://pubmed.ncbi.nlm.nih.gov/35412922/

3. Donut-shaped Corneal Allogeneic Intrastromal Segment as an Alternative to Deep Anterior Lamellar Keratoplasty in Advanced Keratoconus. Coscarelli S, Coscarelli SP, Torquetti L. Cornea. 2024 May 1;43(5):658-663. doi: 10.1097/ICO.0000000000003456. Epub 2024 Jan 5. PMID: 38178305 - https://pubmed.ncbi.nlm.nih.gov/38178305/

4. Femtosecond Laser- Assisted Customized CAIRS In Keratoconus Patients After ICRS Explantation: A Prospective Case Series: Customized CAIRS in post ICRS extrusion keratoconus patients. Susanna BN, ForsetoMD AS, SilveiraMD AM, Santhiago MR, Susanna FN, Pereira NC. J Cataract Refract Surg. 2024 Dec 16. doi: 10.1097/j.jcrs.0000000000001600. Online ahead of print. PMID: 39680687 - https://pubmed.ncbi.nlm.nih.gov/39680687/

5. Mazzotta C, Zagari M, Bona G, Ponzin D, Awwad ST, Torres-Netto EA, Hafezi F, Jacob S. Crosslinked All-Femtosecond Laser-Cut Corneal Allogenic Intracorneal Ring Segments (AFXL CAIRSs): Pilot Ex Vivo Study and Report of First Two Cases Performed in Italy. J Clin Med. 2024 Sep 27;13(19):5771. doi: 10.3390/jcm13195771. PMID: 39407831; PMCID: PMC11476447.

Case reports:

6. Online case report - Case Report of Corneal Allogenic Intrastromal Ring Segment (CAIRS) in a Keratoconic Eye Florencia Yeh, OD, FAAO, FSLS, Pierce Kenworthy, OD, FAAO, Robert E. Fintelmann, MD, FACS https://clinicalinsightsineyecare.scholasticahq.com/article/89455
7. Corneal Allogenic Intrastromal Ring Segments as a Therapeutic Method for Intrusion of Synthetic Intrastromal Corneal Ring Segment: A Case Report Jeremiah E. Gendy, FICO, MRCSEd, MSc, Yara Bteich, MD, Jad F. Assaf, MD, Karim Barake, and Shady T. Awwad, MD Journal of Refractive Surgery Case Reports, 2023;3(3):e33–e37
8. Polymethylmethacrylate and Corneal Allogenic Intrastromal Ring Segments: Same Cornea, Different Optical Zones Yara Bteich, MD, Perla Ibrahim, MD, Jeremiah Gendy, FICO, MRCSEd, MSc, Jad F. Assaf, MD, Karim Barake, and Shady T. Awwad, MD Journal of Refractive Surgery Case Reports, 2023;3(3):e38–e41
9. Corneal Allogenic Intrastromal Ring Segment Implantation for Post-LASIK Ectasia - Timothy S. Kalas, MBBS (Hons I), MMed(OphthSc) and David Gunn, MBBS (Hons I), FRANZCO Journal of Refractive Surgery Case Reports, 2023;3(1):e1–e4

10. Susanna, Bianca N MD1,2,a; ForsetoMD, Adriana S PhD1,3; SilveiraMD, Aline M PhD1,4; Santhiago, Marcony R MD, PhD2; Susanna, Fernanda N MD2; Pereira, Nicolas C MD, PhD1,4. Femtosecond Laser- Assisted Customized CAIRS In Keratoconus Patients After ICRS Explantation:

A Prospective Case Series: Customized CAIRS in post ICRS extrusion keratoconus patients. Journal of Cataract & Refractive Surgery ():10.1097/j.jcrs.0000000000001600, December 16, 2024. | DOI: 10.1097/j.jcrs.0000000000001600

Technique:

11. Asymmetric All-Femtosecond Laser-Cut Corneal Allogenic Intrastromal Ring Segments. Bteich Y, Assaf JF, Gendy JE, Müller F, Jacob S, Hafezi F, Awwad ST. J Refract Surg. 2023 Dec;39(12):856-862. doi: 10.3928/1081597X-20231018-04. Epub 2023 Dec 1. PMID: 38063823 https://pubmed.ncbi.nlm.nih.gov/38063823/

12. Extended Dehydration of Corneal Allogenic Intrastromal Ring Segments to Facilitate Insertion: The Corneal Jerky Technique. Awwad ST, et al. Cornea. 2023. PMID: 37399565 https://pubmed.ncbi.nlm.nih.gov/37937759/

13. Biologic stromal ring to manage stromal melting after intrastromal corneal ring segment implantation Jarade, Elias MD1,2,3,*; Issa, Mohamad MD1; Chanbour, Wassef MD1; Warhekar, Pramod MD4

14. Trypan blue-assisted corneal allogenic intrastromal ring segment implantation. Jack S Parker, Philip W Dockery, John S Parker. J Cataract Refract Surg2021 . Jan 1;47(1):127.

15. Parker JS, Dockery PW, Jacob S, Parker JS. Preimplantation dehydration for corneal allogenic intrastromal ring segment implantation. J Cataract Refract Surg. 2021 Nov 1;47(11):e37-e39. doi: 10.1097/j.jcrs.0000000000000582. PMID: 34675164.

16. Bowman Layer Marking for Correct Placement of Corneal Allogeneic Intrastromal Ring Segments. Jacob S, Agarwal A, Awwad S, Parker J, Mimouni M, J S. Cornea. 2024 Mar 1;43(3):398-401. doi: 10.1097/ICO.0000000000003431. Epub 2023 Nov 28. PMID: 38315501 - https://pubmed.ncbi.nlm.nih.gov/38315501/

17. Flattening the curve: manual method for corneal allogenic intrastromal ring segment implantation. Parker JS, Dockery PW, Parker JS. J Cataract Refract Surg. 2021 Nov 1;47(11):e31-e33. doi: 10.1097/j.jcrs.0000000000000555. PMID: 33577275 https://pubmed.ncbi.nlm.nih.gov/33577275/

18. Mechleb N, Flamant R, Panthier C, Ghazal W, Dubois M, Gatinel D, Saad A. Technique of multiple corneal allogenic ring segment preparation using femtosecond laser: preclinical study on human corneal grafts. J Cataract Refract Surg. 2024 May 1;50(5):518-522. doi: 10.1097/j.jcrs.0000000000001399. PMID: 38251934.

19. Jacob S. Custom-shaped CAIRS for personalized treatment of Keratoconus. Indian J Ophthalmol. 2025 Jan 1;73(1):3-5. doi: 10.4103/IJO.IJO_2589_24. Epub 2024 Dec 23. PMID: 39723847.
